# Identification of the Karyopherin Superfamily in Maize and Its Functional Cues in Plant Development

**DOI:** 10.3390/ijms232214103

**Published:** 2022-11-15

**Authors:** Lu Jin, Guobin Zhang, Guixiao Yang, Jiaqiang Dong

**Affiliations:** 1 The Key Laboratory of Plant Development and Environmental Adaptation Biology, Ministry of Education, School of Life Sciences, Shandong University, Qingdao 266237, China; 2 College of Agronomy, Shandong Agricultural University, Taian 271018, China

**Keywords:** Karyopherin, Importin α, Importin β, nucleo-cytoplasmic transport, maize, NLS, NES, phytohormone signaling, auxin, root development

## Abstract

Appropriate nucleo-cytoplasmic partitioning of proteins is a vital regulatory mechanism in phytohormone signaling and plant development. However, how this is achieved remains incompletely understood. The Karyopherin (KAP) superfamily is critical for separating the biological processes in the nucleus from those in the cytoplasm. The KAP superfamily is divided into Importin α (IMPα) and Importin β (IMPβ) families and includes the core components in mediating nucleocytoplasmic transport. Recent reports suggest the KAPs play crucial regulatory roles in *Arabidopsis* development and stress response by regulating the nucleo-cytoplasmic transport of members in hormone signaling. However, the KAP members and their associated molecular mechanisms are still poorly understood in maize. Therefore, we first identified seven *IMPα* and twenty-seven *IMPβ* genes in the maize genome and described their evolution traits and the recognition rules for substrates with nuclear localization signals (NLSs) or nuclear export signals (NESs) in plants. Next, we searched for the protein interaction partners of the ZmKAPs and selected the ones with *Arabidopsis* orthologs functioning in auxin biosynthesis, transport, and signaling to predict their potential function. Finally, we found that several *ZmKAPs* share similar expression patterns with their interacting proteins, implying their function in root development. Overall, this article focuses on the Karyopherin superfamily in maize and starts with this entry point by systematically comprehending the KAP-mediated nucleo-cytoplasmic transport process in plants, and then predicts the function of the ZmKAPs during maize development, with a perspective on a closely associated regulatory mechanism between the nucleo-cytoplasmic transport and the phytohormone network.

## 1. Introduction

Eukaryotic cells establish separate functional spaces for transcription and translation in the nucleus and cytoplasm. The nuclear pores and nuclear pore complexes (NPCs) across the nuclear envelope link two cellular compartments for high-efficiency molecular exchange channels [1,2]. Disordered phenylalanine- and glycine-rich nucleoporins (FG-Nups) are distributed in the center of NPCs, serving as a bidirectional permeability gate to restrict arbitrary translocation of macromolecules [3,4]. Ions, metabolites, and signal-independent small molecules diffuse freely through the NPCs; macromolecules such as proteins, RNAs, and some complexes more than ~5 nm or ~40 kDa in size are usually signal-dependent active transport-mediated by a range of nuclear transport receptors (NTRs) [5,6]. An evolutionarily conserved superfamily of soluble receptors is primarily responsible for the nucleo-cytoplasmic transport (NCT) of the macromolecules, known as Karyopherins (KAPs) or Importins (IMPs) [7,8]. The KAPs play central roles in substrate screening and transport via recognition of the specific short-peptide signals displayed on cargos, referred to as nuclear localization signals (NLSs) or nuclear export signals (NESs) [9,10].

Appropriate nucleo-cytoplasmic partitioning of specific proteins is the critical intracellular step for executing downstream physiological functions [11,12]. However, how the intracellular distribution of nuclear proteins is regulated remains incompletely understood. KAPs may act as upstream regulators of the functional components for gene regulation, chromatin modulation, and signal transduction [13,14,15]. Some published reports have demonstrated the pivotal roles of several KAP members in plant growth, reproduction, immunity, stress response, and epigenetic regulation (Table S1). However, the role of only one member of the KAP superfamily in maize has been revealed: its role in mediating the nuclear accumulation of Opaque2 (O2) to promote zein biosynthesis in kernel development [16,17]. In contrast, the other members of the KAP superfamily and their functions in maize are still unknown.

Therefore, this review first identifies seven *IMPα* and twenty-seven *IMPβ* genes in the maize genome, then starts with this entry point to review the evolution traits of the KAP superfamily, the KAP-mediated nucleo-cytoplasmic transport pathway, and the recognition rules for substrates with nuclear localization signals (NLSs) or nuclear export signals (NESs) in plants. Furthermore, we spotlight the regulatory roles of nucleo-cytoplasmic transport in phytohormone signaling and execution. Next, we searched for the protein interaction partners of the ZmKAPs and selected the ones with *Arabidopsis* orthologs functioning in auxin biosynthesis, transport, and signaling to predict their potential function. Lastly, several *ZmKAPs* were observed to share similar expression patterns with their interacting proteins, implying their potential functions in root development.

## 2. A General View of the Karyopherin Superfamily

The Karyopherin superfamily is categorized into Importin α (IMPα) and Importin β (IMPβ) based on structural and functional features [7]. Genome-wide identification of the IMPα or IMPβ families in *Saccharomyces cerevisiae*, *Danio rerio*, *Homo sapiens*, *Mus musculus*, *Arabidopsis thaliana*, and *Solanum tuberosum* has been successively reported [18,19,20,21]. The IMPαs serve as a protein adaptor between cargo and IMPβ1 in the classical nuclear import pathway in yeast and mammals, and most IMPβs can independently mediate nuclear–cytoplasmic transport [22,23]. Generally, IMPβs are divided into importins and exportins, while a few IMPβs perform a dual role in both nuclear import and export, such as ScKAP142/ScKAP122, HsXPO4/HsXPO7, and HsIPO13 in yeast and humans [24,25,26,27,28]. However, these bidirectional receptors are demonstrably undetermined in plants. In addition, the function of some KAPs remains poorly understood in plants (Supplementary Materials Table S1).

### 2.1. Evolution of the Karyopherins

**Figure 1:**
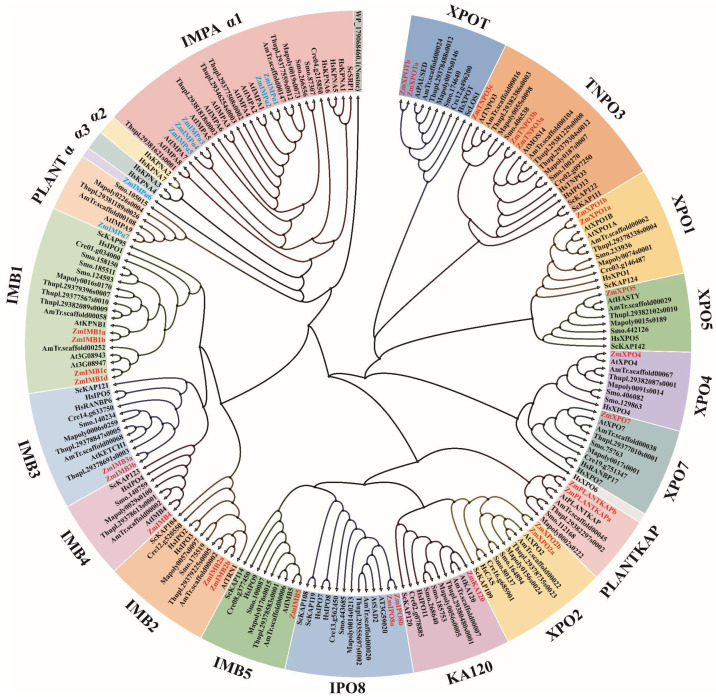
Phylogenetic tree of the Karyopherin superfamily. Based on validated members of the IMPα and IMPβ families from yeast, humans, and *Arabidopsis*, each protein sequence was used as a query to perform BLASTP searches in Phytozome v13 (https://phytozome-next.jgi.doe.gov/ (accessed on 4 October 2022)), NCBI (https://www.ncbi.nlm.nih.gov/ (accessed on 4 October 2022)), and MaizeGDB (https://www.maizegdb.org/ (accessed on 4 October 2022)), remove the non-representative splicing forms of the same gene locus, and confirm sequences of non-redundant candidates by phylogenetic analysis with the homologous series of the other species. *Saccharomyces cerevisiae* (Sc), *Homo sapiens* (Hs), *Chlamydomonas reinhardtii* (Cre), *Marchantia polymorpha* (Mapoly), *Selaginella moellendorffii* (Smo.), *Thuja plicata* (Thupl.), *Amborella trichopoda* (AmTr.), *Arabidopsis thaliana* (At), *Zea mays* (Zm); ZmIMPα proteins in blue font and ZmIMPβ in red.

Based on validated members of the IMPα and IMPβ families from yeast, humans, and *Arabidopsis*, each protein sequence was used as a query to perform BLASTP searches against the blue-green algae (*Nostoc*), green algae (*Chlamydomonas reinhardtii*), bryophyte (*Marchantia polymorpha*), pteridophyte (*Selaginella moellendorffii*), gymnosperm (*Thuja plicata*), angiosperm (*Amborella trichopoda*), and maize genomes (Figure 1). For this analysis, the *KAPs* are an ancient gene superfamily existing in all eukaryotes. In blue-green algae, a few sequences referred to as HEAT (Huntingtin, elongation factor 3 (EF3) 1, protein phosphatase 2A (PP2A) 2, and the yeast PI3-kinase TOR1) repeat domain-containing proteins share a low similarity with IMPα and IMPβ, which may suggest the evolutionary source of their unique properties. In eukaryotes, the KAP superfamily is highly conserved from single-celled to multicellular organisms. Among plant species, the PLANTKAP clade in the IMPβ family is unique to embryophyte plants. Analogously, there is also an embryophyte plant-specific group in the IMPα family, and we named this clade PLANTα (Figure 1).

Results ultimately identified seven *IMPαs* and twenty-seven *IMPβs* in maize, named based on their subfamily affiliation (Table 1). In comparison to *Amborella trichopoda* and *Arabidopsis*, the members of maize *KAPs* undergo family expansions, especially in the IMPβ family. The lineage of maize experienced a tetraploidy period combined with two genomes, the Maize1 and Maize2, accompanied by whole genome duplication (WGD) [29,30]. As shown in Supplementary Materials Table S2, sixteen of thirty-four *KAP* genes experienced duplication and retained elements from ancient tetraploid maize genomes. There are fourteen *KAP* genes that may undergo uneven gene loss after WDG. Among the thirty-four *KAP* genes, eighteen genes come from the Maize1 subgenome and twelve genes are from the Maize2 subgenome. In addition, four maize *KAP* genes may be dispersed as duplicate genes.

#### 2.1.1. Importin α

*IMPαs* in animals include three subfamilies designated α1, α2, and α3 [18]. Group α1, found in all eukaryotes, is believed to be the earliest progenitor of *IMPαs* and gave birth to the other two groups, which function in development and differentiation for the evolution of metazoan animals [31,32]. Eight of nine *IMPαs* in *Arabidopsis* belong to subfamily α1, and the remaining one is a non-conventional isoform [20]. Replication events based on group α1 are distinct between animals and plants, which may have taken unique evolutionary paths to bring forth particular clades. *ZmIMPα1-5* and *AtIMPA1-8* are orthologous to *ScSRP1* and *HsKAPNA1/5/6,* belonging to clade α1. *ZmIMPα7* is the ortholog of *AtIMPA9* as the non-conventional isoform. This specific group is also present in other species, except for *Chlamydomonas reinhardtii*. Therefore, we named PLANTα as an embryophyte plant-specific group of the *IMPαs*. In addition, *ZmIMPα6* failed to classify into any group, and it appeared to be another gene duplication.

#### 2.1.2. Importin β

*IMPβs* are a large conserved family in which the number of members varies slightly across eukaryotes, and can be divided into fifteen subfamilies (Figure 1). The *ZmIMPβs* lack the XPO6 subfamily and have a PLANTKAP group without a noticeable difference from other eukaryotic plants. The distribution pattern of *IMPβ* subclasses may be established before the evolutionary expansion of eukaryotes, accompanied by continuous selective pressure leading to a secondary loss of the *IMPβ* orthologs [33]. The lack of the XPO6 subfamily in *Arabidopsis* is likely to be a representative loss event, and an analogous situation is available in yeast (XPO4/6/7) [33]. PLANTKAP is a paralogous expansion cluster identified in embryophyte-specific land plants [34]. It indicates the fifteen *IMPβ* subfamilies that are conserved in eukaryotes but at the same time accompanied by ortholog expansion or paralog secondary loss. A report shows decreased IMPβ subfamilies during the evolution of the potato genome, but increased homologous genes within the IMB1 and IMB3 subfamilies in *Solanum tuberosum* [21]. Analogous duplication events might have observably promoted the expansion of the composition of *ZmIMPβ* members, especially in the IMB1, XPOT, XPO2, TNPO3, and PLANTKAP subfamilies compared to *HsIMPβs* and *AtIMPβs*.

### 2.2. The Karyopherin-Mediated NCT Pathway

#### 2.2.1. The Classical Nuclear Protein Import Cycle in Yeast and Mammals

The classical nuclear protein import cycle in which IMPα and IMPβ1 cooperate has been well characterized in yeast and mammals [35]. It includes three steps: (I) In the cytoplasm, cargos with classical NLS (cNLS) are recognized by the IMPαs, linking with the IMPβ1 to form an IMPα/β1 heterodimer localized to the nuclear envelope [36]. Then, the IMPβ1 directly interacts with the FG-Nups to facilitate transport of the cargo–IMPα–IMPβ1 complex across the NPCs [37]. (II) Once the imported complex reaches the nucleus, a conformation change triggered by high-affinity RanGTP binding to the IMPβ1 results in the primary dissociation of IMPβ1 from the IMPα-cargo [38]. This irreversible dissociation also influences the conformational change in IMPα itself and accelerates the release of cargo from the IMPα [39]. (III) Lastly, the empty IMPα is recycled by exportin CAS back to the cytoplasm in preparation for the next round of nuclear import [40].

#### 2.2.2. The IMPα- and IMPβ-Mediated Nuclear Transport Pathway in Plants

Although the classical transport cycle has yet to be confirmed in plants, several reports have shown a conservative mechanism of the IMPα/β-mediated nuclear protein import pathway. A bimolecular fluorescence complementation (BiFC) assay shows the interaction between AtKPNB1 and four AtIMPAs (AtIMPA1, AtIMPA2, AtIMPA4, and AtIMPA6) [41]. The exportin AtXPO2/AtCAS can be specifically bound to AtIMPA1, AtIMPA2, AtIMPA3, and AtIMPA4 in yeast two-hybrid (Y2H) analysis [42]. The AtIMPA2 interacts with the N-terminal region of AtXPO5/AtHASTY to mediate its nuclear shuttling from the cytoplasm to the nucleus [43]. Additionally, the vitro nuclear import assay demonstrates that rice IMPα1 can form a complex with mouse IMPβ1 and cNLS cargo [44]. Interestingly, another report shows that AtIMPα can mediate the nuclear accumulation of NLS cargo independent of IMPβ [45]. It implies that IMPα may not only act as a protein adaptor but also possibly independently mediate a unique nuclear import pathway in plants.

#### 2.2.3. The IMPβ-Dependent Nuclear Translocating Pathway

In eukaryotic cells, the IMPβ family dominates the nuclear translocation transport of most proteins and RNAs [46]. These cargos, with distinctive signals, can directly interact with importins or exportins to constitute multiple non-classical transport pathways [47,48]. These parallel pathways share a similar mechanism to the classical nuclear import cycle in their multivalent interaction with the FG-Nups and directional regulation by the Ran (Ras-like nuclear protein) system, as well as their functional redundancy in the transportation of the same cargos [49,50,51]. The IMPαs and the IMPβs are probably evolutionarily related proteins defined by two helical secondary structures, Armadillo-like (Arm) and the HEAT repeats, which provide interaction scaffolds for multiple protein ligands [52,53]. That might lead to differences in protein conformation flexibility between IMPαs and IMPβs that impact their affinities for specific cargos.

## 3. Importin α Family in *Maize* and *Arabidopsis*

### 3.1. Protein Domain Distribution and Gene Expression Profiles of the ZmIMPαs

**Figure 2:**
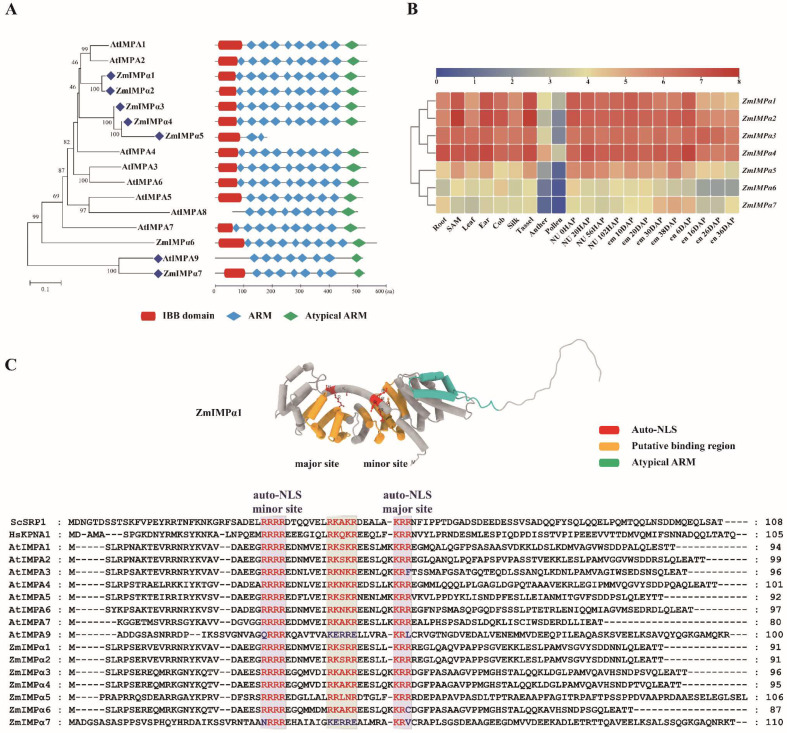
IMPORTINα family in maize and *Arabidopsis*. (**A**) Schematic view of the domains conserved between AtIMPA and ZmIMPα proteins according to Pfam Database (https://pfam.xfam.org/ (accessed on 4 October 2022)) and CCD Tools (https://www.ncbi.nlm.nih.gov/Structure/cdd/wrpsb.cgi (accessed on 4 October 2022)); (**B**) Heat map of the expression pattern of *ZmIMPα* genes, with the expression value calculated by log2 (FPKM). SAM: shoot apical meristem, NU: nucellus, em: embryo, en: endosperm, HAP: Hours after Pollination, DAP: Day after Pollination; (**C**) Signatures of the Importin β binding (IBB) domain of the ZmIMPα1 protein predicted by AlphaFold Protein Structure Database (https://alphafold.com/ (accessed on 4 October 2022)); multiple amino acid sequences of the IBB domain aligned using CLUSTALW, three conserved motifs highlighted in red and rectangle boxes.

Three conserved domains, an N-terminal importin-β-binding (IBB) domain followed by a consecutive ARM repeat region and an atypical ARM repeat at the C-terminal, are predicted by the NCBI and Pfam database of both the AtIMPA and the ZmIMPα proteins (Figure 2A). These conserved domains form a highly similar protein structure in most IMPαs, suggesting their comparable biological function.

The flexible IBB domain is the central zone in recruiting IMPβ1 [54]. In the AtIMPαs, the IBB domain appears to be absent in AtIMPA8 and AtIMPA9. In AtIMPA8 this is due to a reduction in the partial sequence at the N-terminal, while AtIMPA9 seems to have an undefined region. Compared to its homolog in the PLANTα group, ZmIMPα7 contains the IBB domain at the N-terminal. The sequence alignment suggests that the vacant N-terminal of AtIMPA9 may have a similar function to the IBB domain (Figure 2C).

The ARM array and atypical ARM are responsible for cargo loading and CAS binding [55,56]. In the ZmIMPαs, ZmIMPα5 appears to be an incomplete gene copy with a closer kinship to ZmIMPα4. The lack of the multi-ARM repeats region and the atypical ARM may result in nuclear transport function deficiency. Additionally, members in the PLANTα group display a reduced ARM array, which may lead to differences with other isoforms in substrate recognition.

The RNA-seq-based B73 gene expression data from twenty-one tissues at different growth stages were selected and analyzed [57]. As shown in Figure 2B, the *ZmIMPα* genes display a constitutive expression pattern in various organs. *ZmIMPα4* (*Zm00001d009850*) shows high expression in the endosperm (en), which may relate to its role in the transcriptional regulation of storage proteins [16,17]. The expression levels of *ZmIMPα1/2/3/4* are noticeably higher than those of the other isoforms in the IMPα group. A report shows that *AtIMPA9* is highly expressed in the leaves during pathogen infection [58]. The expression profile may imply their potential functional redundancy or differentiation in response to specific ambient cues (Supplementary Materials Figure S1).

### 3.2. Multifunctionality of the IBB Domain

The IBB domain is a critical molecular connector between IMPα and IMPβ, and it is also an ingenious regulator for the activity of IMPα itself. The crystal structure of IMPα in mammalians shows the IBB domain containing an internal NLS that binds to its NLS-binding site and functions as an autoinhibited regulator [39]. The auto-inhibitory action can be displaced by IMPβ1 binding to fulfill its affinity switch to cargos [59]. The alkaline amino acid ^54^KRR^56^ (Lys-Arg-Arg) in the IBB domain of ScSRP1 acts as an auto-inhibitory NLS sequence [60,61]. The mutation of ^54^KRR^56^ does not impact the interaction with IMPβ, but it will lead to the failure of cargo to be released in the nucleus [60]. Additionally, the other two conserved alkaline amino acids in the IBB domain, ^33^RXXR^36^ and ^44^RXXXR^48^ (X for any residue), are likely to significantly affect the binding activity of IMPβ1 [62]. This shows flexible switching roles of the IBB domain in auto-inhibition, interaction with IMPβ, and cargo release.

The protein conformation of the ZmIMPαs displays a similar structure except in ZmIMPα5. As represented in ZmIMPα1, the IBB domain folds back to occupy the NLS-binding surface (Figure 2C). That auto-inhibited state can be switched from closed to open by cooperative binding of the NLS cargo and IMPβ to the IMPα [63]. In the putative IBB domain sequence, three clusters of alkaline amino acids in the AtIMPAs and ZmIMPαs show subtle distinctions or variations (Figure 2C). The first two clusters in the PLANTα groups show distinct features, such as (Q/N) RRR and KERRE. The RRRR cluster is conservative in other IMPαs such as ScSRP1 and HsKPNA1. The RKXKR motif is the primary pattern in group α1 except for ZmIMPα1/2 (RKSRR), suggesting that amino acid R (arginine) at both ends is likely the most conserved residue. The terminal residue of the last cluster is random, e.g., KRX.

Moreover, a recent study in mice shows that a DNA-binding region can be identified in the IBB domain of KPNA2 and characterized to overlap the conserved alkaline amino acid region [64]. This suggests that the IBB domain may act as a common interacting domain for multiple binding partners involved in the functional switching of the transport and non-transport pathways of IMPαs [65].

### 3.3. The ARM Repeat and Classical NLS Recognition

A series of ARM repeats in IMPαs is mainly responsible for cargo loading and releasing by cooperating with the IBB domain [55]. The consecutive stacking ARM repeats generate a superhelical structure and the inner concave surface of the protein provides NLS-binding grooves for the cargos, which include the major and minor binding pockets for recognizing positively charged amino acid clusters in NLSs [66,67]. NLSs with short and regular amino acid clusters generally divide into classical (cNLS) and non-classical NLSs (ncNLS) based on residue composition [68]. The monopartite (MP) and bipartite (BP) motifs are two common types of cNLSs mainly recognized by IMPαs [69]. In addition, the LSD1-type zinc finger motifs possibly act as NLSs bound to the IMPα [70]. That indicates that more potential signals are yet to be discovered and interpreted.

The first identified cNLS in the simian virus 40 (SV40) large T antigen, composed of seven amino acids, was PKKKRKV (Pro-Lys-Lys-Lys-Arg-Lys-Val), identified as an MP-cNLS bound to the major site of the IMPαs [71]. There are five classes of MP-cNLS motifs with a distinctive preference for the major or minor binding sites of IMPαs differently in yeast, plant, and mammals [72,73]. As shown in Table 2, the Class I type MP-cNLS seems to be the most common, while others exhibit flexible variation [74,75,76,77]. AtIMPA1/2/3 can recognize Class I/II/V NLS-containing proteins [76]. The NLS of PIP5K2 is analogous to the Class III consensus motifs and is recognized by AtIMPA6/9 [78].

As shown in Figure 2C, the autoinhibitory sequences in the IBB domain (KRR and RRRR) may act as a BP-cNLS, folding back to occupy the major and minor sites when the IMPα is in an empty state to prevent futile nuclear translocation of unloaded import complexes [79]. In rice, OsIMPAα1 may show binding activity to variable motifs on different proteins, suggesting a mutual co-recognition mechanism in BP-cNLS [80,81]. Additionally, there is more than one NLS displayed on cargo; for example, AtMINIYO has two NLSs that may promote its accumulation in the nucleus [77].

## 4. Importin β Family in *Maize* and *Arabidopsis*

### 4.1. The Characteristic Domains of IMPβ Proteins

**Figure 3:**
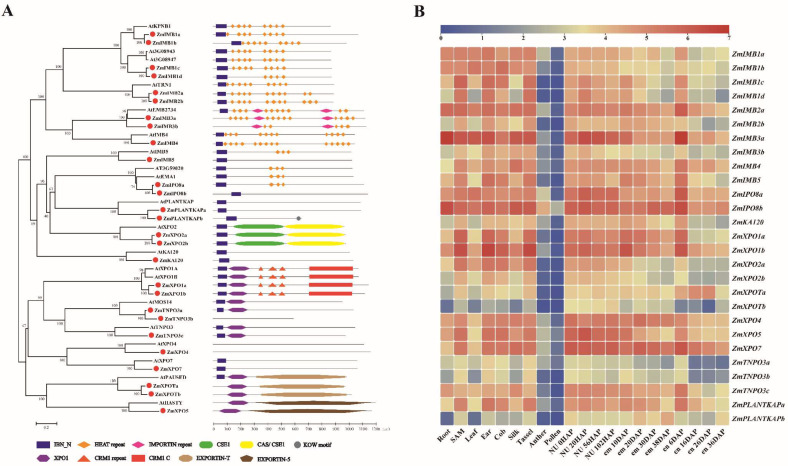
IMPORTINβ family in maize and *Arabidopsis*. (**A**) Schematic representation of the conserved domains between AtIMPβ and ZmIMPβ proteins according to Pfam Database and CCD Tools; (**B**) Heat map of the expression profile of *ZmIMPβ* genes in different tissues, with the expression value calculated via log2 (FPKM). SAM: shoot apical meristem, NU: nucellus, em: embryo, en: endosperm, HAP: Hours after Pollination, DAP: Day after Pollination.

Compared to the high similarity among the IMPα proteins, the IMPβs may represent a more flexible transport receptor family containing various functional domains (Figure 3A). The increased numbers of homologous genes in the IMB1, IMB2, IMB3, XPO2, XPOT, TNPO3, and PLANTKAP subfamilies form a larger family than the AtIMPβs. The conserved domains stay the same in importin and exportin subfamilies between maize and *Arabidopsis*, implying that members of each group hold potential functional resemblances. As shown in Figure 3B, most *ZmKAPβ* genes display a constitutive expression pattern suggesting their indispensable roles in maize growth and development. The homologous genes appear to have differential expression levels in each subfamily, potentially indicating neo- and sub-functionalization of these proteins.

#### 4.1.1. Importin

Four ZmIMB1s with high protein similarity are classed into the IMB1 subfamily. ZmIMB1a and ZmIMB1b appear to be the closest orthologs to AtKPNB1, while ZmIMB1c and ZmIMB1d show higher kinship to the other two ARM repeat superfamily proteins, At3G08943 and At3G08947. The importins contained in the IMB1/2/3/4/5, IPO8, KA120, PLANTKAP and TNPO3 subfamilies independently mediate nuclear import. In *Arabidopsis*, AtKPNB1 and AtSAD2 have shown prominent functions in responses to various abiotic stresses [41,87]. AtTRN1, AtKETCH1, and AtSAD2 have demonstrated different roles in microRNA biogenesis and activity regulation [88,89,90]. Both AtKA120 and AtMOS14 act as modifiers of Suppressor of *npr1-1*, constitutive (SNC1) to affect plant immunity response [91,92]. In yeast and mammals, KAP122 and IPO13 may act as bidirectional receptors [26,28]. The protein domain of the TNPO3 subfamily shares high similarity with the exportins in *Arabidopsis* and maize, which may imply their function in nuclear export, and still needs further verification in plants.

#### 4.1.2. Exportin

The exportins exhibit unique domains in each group and remain highly consistent in *Arabidopsis* and maize. The XPO1 domain is a common feature among the XPO1, XPOT, XPO5, and TNPO3 subfamilies. In the XPO1 subfamily, the CRM1_C domain may contribute to the transition from an extended to a compact conformation in NES–cargo binding [93,94]. A report suggests that the CRM1_C domain in AtXPO1 functions to facilitate virus infection in the nuclear export of viral replicase [95]. Members in the XPO2 subfamily have two related domains, CSE1 and CAS/CSE1, which appear to form a flexible conformation that changes upon cargo binding [96]. XPOT and XPO5 are primarily involved in the nuclear export of multiple RNAs to the cytoplasm [97]. The EXPORTIN-T and EXPORTIN-5 domains are likely to provide the binding pocket for various RNAs [98,99]. Remarkably, however, the link between the protein conformation of the IMBβs and their distinctive cargos is still an open question. This may be inseparable from the function of these conserved domains and still needs further exploration and verification.

### 4.2. The Function of the IBN_N Domain and Ran System

The Importin-beta N-terminal domain (IBN_N) is a typical structural feature at the N-terminal of most IMPβs (Figure 3A). It seems to play a role in cooperation with the Ran system. Several reports show that the residues at the N-terminals of KPNB1, TPNO1, and CSE1 provide the first interactive interface for the Ran protein [37,38,100,101]. The crystal structure of XPO4 in mammals has revealed four distinct Ran-interaction sites, and the N-terminal is in charge of the first Ran-binding site [102]. In *Arabidopsis*, the Ran interacts with the amino terminus in AtHASTY, AtTRN1, and AtMOS14 [83,91,103]. In addition, the IBN_N domain of AtXPO1 appears to support the binding activity of virus protein to impact mosaic virus replication [95].

The IMPβs bound to RanGTP are the direct target regulated by the Ran system [23,38]. In the nucleus, RanGTP binds to IMPβ1 to facilitate the disassembly of the IMPα- β1 cargo [38]. In the cytoplasm, Ran-binding protein 1 (RanBP1) and RanBP2 cooperate with RanGTPase-activating protein 1 (RanGAP1) to hydrolyze RanGTP to RanGDP for releasing IMPβ1 [104,105]. The gradient distribution of the RanGTP/GDP in the nucleus and cytoplasm ensures the proper direction of the nucleocytoplasmic traffic [50]. Therefore, the RanGTP/GDP transformation, the KAP-mediated cargo transport, and the restriction of the NPC complex constitute a multiple-layer control for NCT [106].

### 4.3. The Non-Classical NLS and NES Recognized by Importin β

#### 4.3.1. The Non-Classical NLS

Unlike arginine or lysine residue-enriched cNLSs, only a few ncNLSs or other types of NLSs are structurally characterized and recognized according to their particular IMPβs [68,107]. The PY (proline-tyrosine) motif is a distinguishing feature of the ncNLS that interacts with members of the IMB2 and IMB4 subfamilies [82,108,109]. The PY-NLS has loose sequence motifs in a disordered structure and its overall basic charge is irregular and variable among different cargos [107,110]. The M9 domain of human heterogeneous nuclear ribonucleoprotein A1 (hnRNP A1) with a typical PY-NLS interacts with HsTNPO1 [110]. In *Arabidopsis*, two small RNA-binding proteins, AtGRP7 and AtGRP8, contain an M9-like domain to interact with the ortholog AtTRN1 [83]. The difference in several amino acid residues between the M9 and M9-like domains suggests a discrepancy in the PY-NLS between plants and animals (Table 2). Additionally, the PY motifs seem to function not just in nucleocytoplasmic shuttling. AtIMB4 interacts with the PY motifs in FRA1 kinesin to inhibit its motility and protect protein stabilization in the cytoplasm [82]. Additionally, there are two other types of NLSs, recognized by their designated IMPβs in yeast and human. ScKAP121 and HsIPO5 can bind to a specific IK (isoleucine-lysine-rich)-NLS with a consensus motif K-V/I-X-K-X1–2-K/H/R [111]. HsTNPO3 can mediate the cellular trafficking of SR proteins (serine/arginine-rich proteins) through interaction with the RS (arginine–serine) repeat domain [112]. However, these two analogous NLS are still unknown in plants.

#### 4.3.2. NES

NES is a leucine-rich peptide signal in the nuclear export process, primarily recognized by the exportin XPO1/CRM1 [113]. A set of ten consensus sequence patterns apply to the NES family in animals and plants [114,115]. As shown in Table 2, the NES motifs of zinc finger transcription factor OXS2 members show high conservation in *Arabidopsis*, rice, and maize [84,85]. NES and NLS may coexist in transcription factors such as AtFHY1 and OsWRKY62, suggesting a dynamic nucleocytoplasmic distribution of the nuclear proteins in plant developmental and environmental responses [74,80]. A similar situation also presents itself in plant virus proteins that may facilitate the virus’s replication cycle in plant host cells [86]. Generally, these identified NLSs or NESs are linear targeting signals for IMPαs or IMPβs. In addition, the folded domains in some cargos are likely to bind to IMPβ as well, and that may be related to the particular conformation of the IMPβs [111,116]. However, for other exportins, the more extensive identification signals are still an outstanding problem requiring further elucidation of the potential interaction mechanism.

## 5. Functional Cues of Karyopherins in Hormone Signaling and Plant Development

### 5.1. The Roles of Arabidopsis KAPs in Hormone Signaling

**Figure 4:**
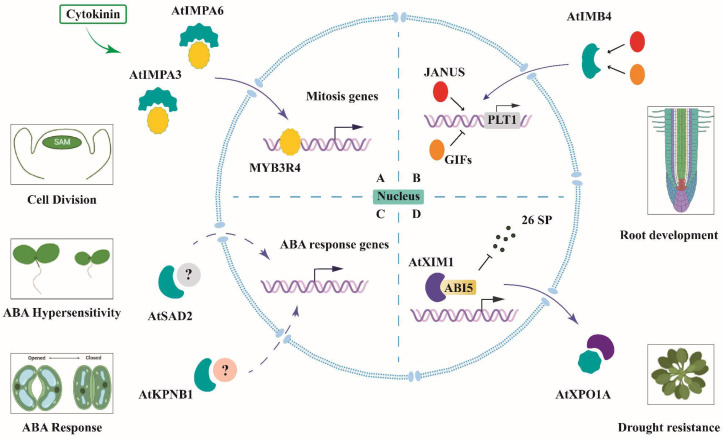
A schematic illustration of *Arabidopsis* KAP-mediated nucleo-cytoplasmic transport in hormone signaling for plant development. (**A**) Cytokinin regulates cell division by promoting nuclear shuttling of transcription factor MYB3R4, mediated by AtIMPA3 and AtIMPA6, in the shoot apical meristem (SAM). (**B**) AtIMB4 mediates the nuclear partitioning of GRF-INTERACTING FACTOR1 (GIF1)/ANGUSTIFOLIA3 and JANUS, which antagonistically regulate *PLETHORA1 (PLT1)* transcription. (**C**) AtSAD2 and AtKPNB1 act as negative regulators in abscisic acid (ABA) signaling. The *atsad2* mutant displays an ABA hypersensitivity response during seed germination and seedling growth. AtKPNB1 is involved in controlling ABA-induced stomatal closure under drought conditions. (**D**) AtXPO1A mediates the nuclear export of a WD40 repeat-containing protein, XIW1 (XPO1-interacting WD40 protein 1), which maintains the stability of ABA INSENSITIVE 5 (ABI5) in the nucleus. The schematic illustration was drawn with BIORENDER (https://biorender.com/ (accessed on 4 October 2022)).

Phytohormones are important in regulating transcriptional networks in plant growth and environmental adaption [117]. Recently, some encouraging progress has been made in understanding the regulatory roles of the *Arabidopsis* KAPs in plant hormone pathways, and a schematic illustration is shown in Figure 4. Of note, this motivates a stepwise progression towards new insight into the more regulatory components in the phytohormone network.

#### 5.1.1. AtIMB4 and PLT1-Mediated Root Development

PLETHORA (PLT) family members encoding AP2 class transcription factors depend on auxin response [118]. Auxin-induced PLTs form a gradient to control the location of the stem cell region and root meristem size. [119]. AtIMB4 is a positive regulator in root meristem size [120]. It is involved in transcriptional regulation for the *PLT1* gene by mediating the nuclear accumulation of two antagonistic cargos, JANUS and GIF1 [120].

#### 5.1.2. AtIMPA3/6 and Cytokinin-Activated Cell Division in Shoot Apical Meristem

Myb-domain protein 3R4 (MYB3R4) transcription factor is highly expressed in the shoot apical meristem and enriched in the dividing cells to activate the expression of the cell cycle genes during mitosis [121]. Generally, MYB3R4 is mainly localized in the cytoplasm, and AtIMPA3 acting together with AtIMPA6 mediates its rapid nuclear accumulation triggered by cytokinin at the G2/M transition [121].

#### 5.1.3. AtIMPβs and ABA Signaling in Response to Abiotic Stress

There are three IMPβs, AtSAD2, AtKPNB1, and AtXPO1A, shown to be involved in ABA signaling in responses to abiotic stress. AtSAD2 is initially found in the abscisic acid (ABA) hypersensitivity response during seed germination and seedling growth as a negative regulator of ABA sensitivity, suggesting its potential function in ABA signaling [122]. AtKPNB1 also acts as a negative regulator at early steps in ABA signaling, and it might play an essential role in controlling ABA-induced stomatal closure under drought conditions [41,123]. Conversely, AtXPO1A mediates the nuclear export of a WD40 repeat-containing protein XPO1-interacting WD40 protein 1 (XIW1) [124]. In the nucleus, XIW1 interacts with the key transcription factor ABA INSENSITIVE 5 (ABI5) in the ABA signaling pathway to maintain its stability and further positively regulate the ABA response [124].

### 5.2. The Predicted Interacting Protein of the ZmKAPs Involved in the Auxin Pathway

Reports in *Arabidopsis* suggest that the Karyopherin-mediated nucleocytoplasmic shuttling of signal molecules is the critical link to the hormone signal transduction chain [120,121,124]. However, more signal elements remain to be discovered for obtaining a better understanding of the role played by KAPs in the phytohormone network, especially for corn growth and development. Therefore, to understand the functional cues of the ZmKAPs, we explored the putative interacting proteins using plant.MAP and STRING database [125,126]. As the function of most proteins in maize is not yet studied, we selected their orthologs in *Arabidopsis* involving auxin biosynthesis, transport, and signaling to discuss their potential functionality links (Table 3).

#### 5.2.1. Auxin Biosynthesis

The tryptophan (TRP)-dependent/indole-3-pyruvic acid (IPyA) pathway in two-step auxin biosynthesis has been well characterized to finely tune the local auxin synthesis in response to various internal development cues and external stimuli [127]. AtIMPA1/2/3 play redundant roles in the nuclear import of LHP1 and are necessary for flowering regulation [76]. In auxin biosynthesis, LHP1 links SUPERMAN (SUP) and polycomb repressive complex 2 (PRC2) to repress the expression of *YUC1* and *YUC4* genes and fine-tune local auxin signaling in the floral meristem [128]. However, another report shows that LHP1 is a positive regulator for *YUC* genes in leaves, suggesting its complicated roles in auxin biosynthesis in different tissues or at different developmental stages [129]. Chromatin remodeling factors CHR11 and CHR17 and *Arabidopsis* DEAH-box splicing factor PRP16 are predicted to be the downstream targets for IMPα1/2/3/4 and IPO8 (Table 3). CHR11 and CHR17 form a complex with AGAMOUS (AG) at the proximal region of the *YUC4* promoter to control its chromatin accessibility for transcription regulation in the floral meristem [130]. The expression of the *YUC4* gene is regulated via alternative splicing to generate two splice variants with tissue-specific distributions [131]. The mutation of *PRP16* disturbs the expression trait of *YUC4* transcript variants in seedlings and cauline leaves, as well as the expression of several other genes involving auxin biosynthesis [132].

#### 5.2.2. Auxin Transport

Intercellular directional auxin transport depends on PIN-FORMED (PIN) auxin efflux transporters [133]. IMPα1/2/3/4 and IMB1 seem to be responsible for the nuclear import of NAP1-related protein NRP1/2 and nuclear RNA polymerase II subunit NRPB2, which may influence the expression and location of PIN proteins (Table 3). Histone chaperones NRP1 and NRP2 are recruited at the *PIN1* locus for local chromatin modulation and coordinate with the *Arabidopsis* chromatin-remodeling factor INOSITOL AUXOTROPHY 80 (AtINO80) to control the size of meristem inflorescence [134]. NRBP2 is the second-largest subunit of RNA pol II required in mRNA and non-coding RNA biosynthesis [135]. The root tips of the *nrpb2-3* mutant display strongly decreased expression and positioning of the PIN1/2/3 proteins, which may change local auxin levels, resulting in *WUSCHEL-RELATED HOMEOBOX 5 (WOX5)* ectopic expression in the root apical meristem (RAM) [136]. In addition, PRP16 seems to regulate the expression of most *PIN* genes in flowers or seedlings and influences the proper subcellular localization of PIN1 in roots as well [132].

#### 5.2.3. Auxin Signaling

The SKP1/CULLIN1/F-BOX(SCF)-type E3 ubiquitin ligase complex is critical for auxin perception and signaling in the nucleus [137]. The F-box proteins TRANSPORT INHIBITOR RESPONSE 1/AUXIN SIGNALING F-BOX (TIR1/AFB), as auxin receptors, mediate the degradation of Auxin/Indole-3-Acetic Acid (AUX/IAA) transcriptional repressors via 26S proteasome (26SP) to release AUXIN RESPONSE FACTOR (ARF) transcription factors, leading to transcriptional reprogramming [138].

HDA6 is a negative regulator of gene expression, and AtXPO1A functions as an anti-silencing factor by mediating the nucleocytoplasmic partitioning of HDA6 [139]. HDA6 and HDA9 may act synergistically in the auxin signaling pathway to regulate valve cell elongation, and they exhibit functional redundancy in the expression of the *ARF4* gene in silique valves [140]. The ortholog *HDA108* (*Zm00001d050139*) is essential for maize development, and the mutant exhibits defects in fertility due to altered ear and tassel growth and microgametogenesis in the anthers [141].

IMPα, IMB1, IMB3 and XPO1 appear to interact with HSP90, CAND1, CSN4, and UBP14 proteins, which may be involved in the regulation of the SCF complex (Table 3). HSP90 acts as a chaperone of TIR1 to facilitate its nuclear localization and positively regulates its auxin receptor function in the nucleus [142,143]. Increased temperature promotes HSP90-mediated rapid nuclear accumulation of TIR1, suggesting its role in integration between temperature and auxin signaling [144]. CAND1 is likely to function in the assembly and disassembly cycles of the SCF complex through its interactions with CULLIN1 (CUL1) to regulate SCF^TIR1^ activity [145]. The COP9 signalosome (CSN), composed of eight subunits (CSN1-8), is a conserved nuclear protein complex required for the dynamic modification of cullin [146]. The *csn* mutant exhibits impaired auxin responses, which may be related to SCF^TIR1/AFBs^-mediated protein degradation [147]. CSN4 is involved in the control of adventitious root (AR) formation and modulates the activity of CUL1 by affecting de-neddylation for CUL1-NEDD8 [148]. UPB14 acts on the turnover of cellular proteins via 26SP-mediated degradation and is likely to function with TIR1, ARF7, and AUX1 in auxin signaling [149]. A reduction in UPB14 activity results in delayed lateral root primordium (LRP) initiation and impaired lateral root growth, which may be related to the stabilization of the AUX/IAA repressor proteins in the mutant [149,150].

IMB3, IMB4, IPO8 and XPOT are predicted to be potential interaction factors for tRNA-specific methyltransferase TRM4B (Table 3). TRM4B mediates posttranscriptional methylation of RNA cytosine residues to 5-methylcytosine (m5C), including tRNAs, mRNAs, and noncoding RNAs [151]. It promotes the m5C modification of *SHORT HYPOCOTYL 2 (SHY2)* and *INDOLEACETIC ACID-INDUCED PROTEIN 16 (IAA16)* mRNA and plays a positive role in mRNA stability in root development [152]. Chromatin remodeling protein PKL and WD40-containing protein PRL1 may serve as the interaction targets of the IMPαs and IMB1s (Table 3). PKL interacts with RETINOBLASTOMA-RELATED 1 (RBR1) to serve as a transcriptional repressor of *LATERAL ORGAN BOUNDARIES-DOMAIN 16 (LBD16),* which functions in the symmetric division of lateral root (LR) founder cells [153,154]. The suppression of the PKL–RBR1 complex may be relieved from the *LBD16* promoter by the IAA14/ARF7/ARF19 signaling pathway to facilitate LR formation [154]. *PRL1* encodes a nuclear WD40 protein that has a pleiotropic effect on sugar and several hormone responses and is necessary for the activity of the root stem cell niche and maintenance of the meristem size [155,156]. *PRL1* has cell- and tissue-specific expression traits in RAM during primary root growth and appears to configure *WOX5* expression in the quiescent center (QC) to act as an upstream regulator of the PLT1/PLT2 dependent pathway [156].

Additionally, IMPα1/2/3/4, IMB3, and IMB4 appear to interact with another WD40 protein, PCN, and a regulatory component of 26SP. The *PCN* gene encodes a nuclear WD40 protein that may integrate auxin signaling into the organization and maintenance of apical meristems [157]. It appears to coordinate with BODENLOS (BDL) and TOPLESS (TPL) to mediate the repression of *MONOPTEROS (MP)* genes and other targets in the auxin signaling pathway [157]. The regulatory particle AAA-ATPase 5a (RPT5a) is a 26SP subunit that possibly facilitates substrate recognition and unfolding [158,159]. In the *rpt5a* mutant, drastically aberrant auxin and cytokinin responses in roots suggest a role of RPT5a in adjusting the auxin/cytokinin signaling balance to maintain RAM morphology under high boron stress [160].

### 5.3. Expression Profiles of ZmKAPs and Corresponding Interaction Partners in Root Development

**Figure 5:**
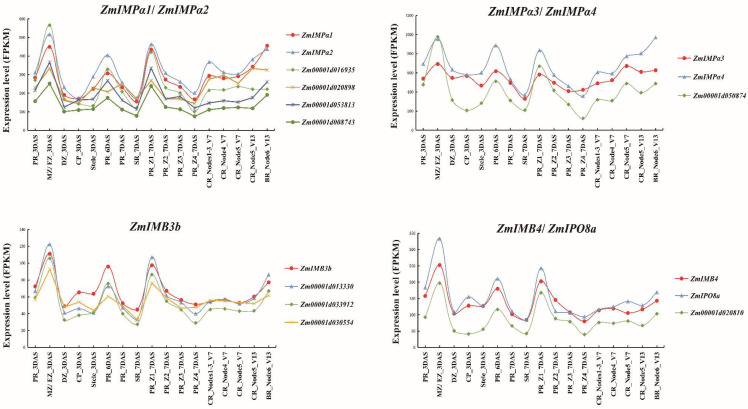
Gene expression profiles of *ZmKAPs* and interacting partners in the root. PR: Primary Root, MZ: Meristem Zone, EZ: Elongation Zone, DZ, Differentiation Zone, CP: Cortical Parenchyma, SR: Seminal Roots, Z1: Zone 1(root tips region), Zone 2 (from the end of Z1 to the point of root hair or lateral root initiation), Zone 3 (lower half of differentiation zone); Zone 4 (upper half of differentiation zone), CR: Crown Roots, BR: Brace Roots, DAS: Day After Sowing, V: Vegetative.

Several orthologs of interaction partners have shown regulatory roles in root development. To gain additional insight into the potential function of ZmKAPs and correlated interacting partners, we searched for their detailed gene expression patterns in roots through RNA-seq based B73 gene atlas data [161]. Figure 5 shows that seven candidates have similar temporal–spatial expression profiles to those of their putative interacting *ZmKAP* genes.

In *Arabidopsis*, the *NRP1/2* double mutant displays a smaller meristem and shorter root than the wild type [134]. *Zm00001d050874/ZmNAP1* and *Zm00001d016935/ZmNFA104* are orthologs of AtNRP1 and -2 that show high transcription levels in the primary roots and the root tip region. The expression level of *ZmIMPα3/4* is the same as that of *ZmNAP1*, and that of *ZmIMPα1/2* is the same as that of *ZmNFA104*. In maize, the *Zm00001d020898/ZmHSP4* gene has upregulated expression induced by heat stress [162]. The *Arabidopsis* HSP90 affects temperature-mediated root and hypocotyl growth through modulating the auxin response [144]. *ZmHSP4* shows high expression levels in primary roots and crown roots, and *ZmIMPα1/2* may present co-expression patterns with *ZmHSP4* during crown root development. OsCAND1 is a regulator of the G2/M transition for meristem cells involved in the emergence of crown root primordia [163]. In maize, the ortholog of *CAND1*, *Zm00001d053813*, exhibits the same expression pattern as *ZmIMPα1/2* in root development.

Analogously, *Zm00001d008743*, *Zm00001d020810*, *Zm00001d013330*, *Zm00001d033912*, and *Zm00001d030554* have high expression levels in the primary roots and the root tip region, which may be closely related to the root meristem zone. Zm00001d020810 appears to interact with more than one ZmKAP, while *ZmIMB4* and *ZmIPO8a* exhibit a more similar transcriptional trend to the *UPB14* ortholog in maize. *ZmIMB3b* seems to have the same expression profile in roots as the other three interaction partners. In addition, Zm00001d030554 is the ortholog of the nucleolus localization protein APUM23, and the mutation of *APUM23* displays reduced and mislocalized auxin maxima within the root tips, suggesting its potential role in auxin homeostasis maintenance [164].

## 6. Conclusions and Perspectives

The KAP-mediated nucleo-cytoplasmic transport of biomacromolecules is the core link in organizing genome activities and triggering downstream cell behaviors. The KAP superfamily and their regulatory mechanisms are highly conserved among eukaryotes and display critical roles in various intracellular biological processes with indispensability in plant growth and development (Supplementary Materials Table S1). However, the KAP superfamily in corn has yet to be studied. Hence, identifying the ZmKaps is essential for understanding new genetic regulatory mechanisms in maize biology. The comparable sub-familial distribution and functional features between maize and *Arabidopsis* suggest their potential similarity in biological functions and cargo recognition mechanisms (Figure 1). Meanwhile, the expanded number of members in the ZmIMB1, ZmIMB2, ZmIMB3, ZmPLANTKAP, ZmXOP2, ZmTNPO3 and ZmXPOT subfamilies may link to the more complex cellular activities in the physiological environment (Figure 3). The proper nucleo-cytoplasmic partitioning of nuclear proteins is a vital mechanism in the plant signaling pathway, including the members of various hormone signal transduction chains [11]. In searching for the interaction partners of ZmKAPs, we obtained some function cues of ZmKAPs in the auxin pathway (Table 3). Although these cues are enlightening, these potential actors still need to be further explored and investigated in maize.

Considering some transient protein–protein interactions in cells is likely far beyond what the database describes; more interaction partners of ZmKAPs and dynamic transport mechanisms remain to be uncovered in the hormone signal transduction chain. Additionally, how to transport some low-stability proteins or cargos lacking nuclear localization signal motifs remains to be illustrated. For example, the F-BOX protein TIR1 lacks an NLS, and HSP90 serves as its chaperone to function in the folding of the nascent protein and promote its nuclear localization [139]. That is probably one of the nucleo-cytoplasmic transport modes, whereas the vast majority of the regulatory networks of phytohormone-related specific transcription factors remain yet unknown. In other respects, KAPs exhibit multifunction beyond the transport receptors in maintaining protein stability, epigenetic regulation, and miRNA processing and movement [43,82,165,166]. That will contribute to a deep understanding of the functional characteristics in the ZmKAP superfamily. In the future, based on the use of the predicted KAP information to build up a mutant library via reverse genetics techniques such as the CRISPR/CAS9 system, these are all meaningful subjects that warrant additional exploration in maize growth and development.

## Figures and Tables

**Table 1:** List of putative Karyopherin gene family members in *Zea mays*.

	Gene Name ^a^	Locus ID ^b^	Chromosomal Location ^c^			Transcript ID	Putative Proteins ^d^		
			Chr	Chr_start	Chr_end		Length (aa)	MW (kDa)	Subcellular Location
IMPα	ZmIMPα1	Zm00001d008345	8	5938159	5944491 (−)	T001	527	57.85	Nucleus/Cytoplasm
	ZmIMPα2	Zm00001d040274	3	35350411	35356233 (+)	T001	529	57.95	Nucleus/Cytoplasm
	ZmIMPα3	Zm00001d037606	6	131468248	85071126 (−)	T001	528	58.13	Nucleus/Cytoplasm
	ZmIMPα4	Zm00001d009850	8	85065908	131476305 (−)	T005	529	58.20	Nucleus/Cytoplasm
	ZmIMPα5	Zm00001d040153	3	29316628	29318539 (+)	T004	183	20.42	Nucleus/Cytoplasm
	ZmIMPα6	Zm00001d022536	7	179671127	179674969 (+)	T008	568	61.71	Nucleus
	ZmIMPα7	Zm00001d008640	8	15537598	15544131 (+)	T002	526	56.54	Nucleus/Cytoplasm
IMPβ	ZmIMB1a	Zm00001d030694	1	153742904	153749377 (+)	T002	1074	116.51	Nucleus/Cytoplasm
	ZmIMB1b	Zm00001d041556	3	127112005	127118515 (−)	T002	987	107.94	Nucleus/Cytoplasm
	ZmIMB1c	Zm00001d038021	6	145393970	145398983 (−)	T001	879	96.77	Nucleus/Cytoplasm
	ZmIMB1d	Zm00001d010512	8	118588081	118591573 (−)	T001	876	96.15	Nucleus/Cytoplasm
	ZmIMB2a	Zm00001d002936	2	27303853	27322287 (+)	T010	891	98.80	Nucleus/Cytoplasm
	ZmIMB2b	Zm00001d026696	10	150180000	150204009 (+)	T005	890	98.86	Nucleus/Cytoplasm
	ZmIMB3a	Zm00001d021893	7	165532355	165542662 (−)	T002	1126	123.26	Nucleus/Cytoplasm
	ZmIMB3b	Zm00001d033632	1	269308497	269321829 (+)	T008	1132	123.78	Nucleus/Cytoplasm
	ZmIMB4	Zm00001d028511	1	37580598	37594480 (−)	T008	1047	114.94	Nucleus/Cytoplasm
	ZmIMB5	Zm00001d045725	9	35215354	35239296 (−)	T001	1028	113.35	Nucleus envelope/Cytosol
	ZmIPO8a	Zm00001d050526	4	96801958	96828810 (+)	T008	1145	128.01	Nucleus envelope/Cytosol
	ZmIPO8b	Zm00001d016479	5	164246013	164266562 (+)	T001	1036	131.22	Nucleus envelope/Cytosol
	ZmKA120	Zm00001d007225	2	225128676	225140701 (+)	T019	1115	116.49	Nucleus/Cytoplasm
	ZmXPO1a	Zm00001d012815	5	776419	787466 (+)	T022	1151	132.30	Nucleus envelope/Cytosol
	ZmXPO1b	Zm00001d034914	1	305341236	305352529 (−)	T037	1122	128.54	Nucleus envelope/Cytosol
	ZmXPO2a	Zm00001d033764	1	272997605	273005246 (+)	T002	981	108.32	Nucleus/Cytoplasm
	ZmXPO2b	Zm00001d013417	5	10817793	10829003 (+)	T005	982	108.52	Nucleus/Cytoplasm
	ZmXPOTa	Zm00001d022125	7	170837895	170846340 (+)	T002	978	107.96	Nucleus/Cytoplasm
	ZmXPOTb	Zm00001d006845	2	217770387	217778785 (+)	T005	1024	113.22	Nucleus/Cytoplasm
	ZmXPO4	Zm00001d032704	1	235324863	235346931 (−)	T036	1165	129.84	Nucleus/Cytoplasm
	ZmXPO5	Zm00001d009270	8	49685540	49721525 (+)	T001	1175	130.20	Nucleus/Cytoplasm
	ZmXPO7	Zm00001d037100	6	112267718	112290870 (+)	T051	1067	121.03	Nucleus/Cytoplasm
	ZmTNPO3a	Zm00001d052632	4	195421971	195454136 (+)	T005	1038	114.09	Cytoplasm
	ZmTNPO3b	Zm00001d014033	5	29806869	29825510 (−)	T001	564	62.27	Cytoplasm
	ZmTNPO3c	Zm00001d032699	1	235073263	235106928 (−)	T030	981	109.64	Cytoplasm
	ZmPLANTKAPa	Zm00001d048628	4	1742938	1750365 (+)	T001	1092	120.63	Nucleus envelope/Cytosol
	ZmPLANTKAPb	Zm00001d019335	7	28400881	28407780 (−)	T004	655	73.54	Nucleus envelope/Cytosol

^a^ Name refers to systematic designation among members of the Karyopherin family applied to *Zea mays* based on homology against *Arabidopsis thaliana* and *Homo sapiens*; ^b^ Gene accession number in maizeGDB (MAIZE GENETICS AND GENOMICS DATABASE); ^c^ Chromosomal location of the *ZmIMPα* and *ZmIMPβ* genes based on the Zm-B73-REFERENCE-GRAMENE (V4.0); ^d^ Basic physicochemical properties of the putative ZmIMPα and ZmIMPβ proteins, and subcellular location predicted by UniProt (https://www.uniprot.org/ (accessed on 4 October 2022)).

**Table 2:** Classification of NLSs and NESs recognized by KAPs in plants.

Type	Consensus Motifs	Cargo	Sequence	NTR	Source
MP-cNLS	Class I— KR (K/R) R or K (K/R) RK	AtFHY1/AtFHL	^40^ **KKRK**	AtIMPA1	*Arabidopsis* [74]
		AtPARP2	^48^ **KRKR**	AtIMPA2	*Arabidopsis* [75]
		AtLHP1	^173^R**KRKR**K	AtIMPA1/2/3	*Arabidopsis* [76]
		AtMINIYO	^253^KLK**KRRK**	AtIMPA4	*Arabidopsis* [77]
	Class II— (P/R) XXKR (^DE) (K/R)	AtVRN1	^173^PTPTPKI**PKKRGR**KKKNADPE	AtIMPA1/2/3	*Arabidopsis* [76]
	Class III— KRX (W/F/Y) XXAF	AtPIP5K2	^239^AT**RKR**SSVDSG**A**GSLTGEKIFPRIC	AtIMPA6/9	*Arabidopsis* [78]
	Class IV— (R/P) XXKR (K/R) (^DE)	–	–	–	*–*
	Class V— LGKR (K/R) (W/F/Y)	VQ-protein	^92^LG**LGKRK**RGPGVSGGKQTKRRSR	AtIMPA1/2/3	*Arabidopsis* [76]
BP-cNLS	Class VI— KRX10–12K(KR) (KR) or KRX10–12K(KR) X (K/R)	AtMINIYO	^1401^**RKR**–^1414^**RYKK**,	AtIMPA4	*Arabidopsis* [77]
		OsWRKY62/OsWRKY76	^8^**RK**–^36^**KKK**	OsIMPα1	*Oryza Sativa* [80]
		OsCOP1	^294^**RKKR**–^312^**KRR**	OsIMPα1b	*Oryza sativa* [81]
		ZmOpaque2	^230^**RKRK**–^241^**RRSRYRK**	OsIMPα1b ZmIMPα4	*Oryza sativa* [81], *Zea mays* [16]
PY-NLS	(basic/hydrophobic) Xn— (R/H/K) (X)2–5 PY	AtFRA1	^311^**KKRK**–^320^**PY**	AtIMB4	*Arabidopsis* [82]
	M9-like domain	AtGRP7	^112^SG**G**GGSYGGG**GGR**REGG**G**G**Y**SG	AtTRN1	*Arabidopsis* [83]
Other NLS	Zinc finger motifs	PsLSD1	^7^**CNGCRNMLLYPRGATNVCCALC**– ^46^**CGGCRTLLMYTRGATSVRCSCC**– ^84^**CANCRTTLMYPYGAPSVKCAVC**	AtIMPA1	*Pisum sativa* [70]
NES	Φ-X2–3-Φ-X2–3-Φ-X-Φ	OXS2	^699^**L**EAW**I**EQ**M**Q**L**/**L**GAL**L**EQ**M**Q**L**	-	*Arabidopsis* [84,85]
		AtFHY1	^54^ **LLPL**	AtXPO1	*Arabidopsis* [74]
		OsWRKY62	^308^**V**DQ**I**PH**I**P**V**	AtXPO1	*Oryza Sativa* [80]
		CMV 2b	^79^**L**-^85^**L**-^87^**L**	AtXPO1	*Mosaic Virus* [86]

cNLS: classical nuclear locational signals. MP: monopartite, BP: bipartite, PY: Proline-Tyrosine, NES: nuclear export signals, NTR: nuclear transport receptor, X: any amino acid, ^D/E: any amino acid except Asp or Glu, Φ: for Leu/Val/Ile/Phe/Met. FHY1: FAR-RED elongated hypocotyl 1, FHL: FHY1-like, PARP: poly (ADP-Ribose) polymerase, LHP1: like heterochromatin protein 1, VRN1: vernalization1, PIP5K2: phosphatidylinositol 4-phosphate 5-kinase 2, VQ-protein: VQ motif-containing protein, COP1: photomorphogenic 1, FRA1: fragile fiber 1, GRP7: glycine-rich RNA-binding protein, OXS2: oxidative stress 2, CMV 2b: cucumber mosaic virus 2b.

**Table 3:** Predicted interacting protein of the ZmKAPs.

NTR	Putative Interactor in *Maize*	Interactive Score	Ortholog of the Putative Interactor in *Arabidopsis*	
			Name	Gene ID
ZmIMPα1/2/3/4 (P, S)	Zm00001d009312	P-0.208, S-0.582	CHR11	AT3G06400
	Zm00001d040831		CHR17	AT5G18620
ZmIMPα1/2/3/4 (S)	Zm00001d014449	S-0.781	LHP1	AT5G17690
ZmIMPα1/2/3/4 (P)	Zm00001d050874	P-0.242	NRP1	AT5G17690
	Zm00001d016935		NRP2	AT1G74560
ZmIMB1c/d (P)	Zm00001d033218	P-0.333	NRPB2	AT4G21710
	Zm00001d013683			
ZmIMPα1/2 (P)	Zm00001d020898	P-0.631	HSP90.2	AT5G56030
	Zm00001d031332			
ZmIMPα1/2 (P), ZmXPO1 (S)	Zm00001d053813	P-0.208, S-0.582	CAND1	AT2G02560
ZmIMPα1/2/3/4 (P), ZmIMB1 (P)	Zm00001d028143	P-0.243, P-0.363	CSN4	AT5G42970
ZmIMPα1/2 (P), ZmIMB3 (P)	Zm00001d008743	P-0.299, P-0.255	UBP14	AT3G20630
ZmIMPα1/2/3/4 (P), ZmIMB1c/d (P)	Zm00001d045109	P-0.299, P-0.303	PKL	AT2G25170
ZmIMPα4 (S)	Zm00001d033309	S-0.421	PRL1	AT4G15900
ZmIMB3, ZmIMB4 (S), ZmIPO8 (S), ZmXPOT (S)	Zm00001d020810	S-0.716, S-0.640, S-0.505	TRM4B	AT2G22400
ZmIMPα1/2/3/4 (P), ZmXPO1/5 (S)	Zm00001d050139	P-0.270, S-0.805	HDA6	AT5G63110
ZmIMB3, ZmIMB4 (S)	Zm00001d013330	S-0.639	PCN	AT4G07410
	Zm00001d033912			
ZmIPO8 (S)	Zm00001d006459	S-0.655	PRP16	AT5G13010
ZmIMB3, ZmIMB4 (S)	Zm00001d030554	S-0.489	APUM23	AT1G72320
ZmIMPα1/2/3/4 (P)	Zm00001d037481	P-0.231	RPT5A	AT3G05530
	Zm00001d018409			

(S) for Data analysis from STRING (https://cn.string-db.org/ (accessed on 4 October 2022)), (P) for data analysis from plant.MAP (http://plants.proteincomplexes.org/ (accessed on 4 October 2022)), (P, S) for Data from both STRING and plant.MAP databases. NTR: Nuclear transport receptor, CHR11/17: CHROMATIN REMODELING 11/17, LHP1: LIKE HETEROCHROMATIN PROTEIN 1, NRP1/2: NAP1-RELATED PROTEIN 1/2, NRPB1/2: Nuclear RNA polymerase II (RNA Pol II) subunit 2, HSP90: HEAT SHOCK PROTEIN 90, CAND1: Cullin-Associated and Neddylation-Dissociated, CSN4: CONSTITUTIVE PHOTOMORPHOGENIC9 (COP9) signalosome subunit 4, UBP14: UBIQUITIN-SPECIFIC PROTEASE14, PKL: PICKLE, PRL1: Pleiotropic Regulatory Locus 1, TRM4B: tRNA-specific methyltransferase 4B, HDA6: Histone deacetylase 6, PCN: POPCORN, PRP16: pre-mRNA-processing factor 16, APUM23: *Arabidopsis* Pumilio 23, RPT5a: Regulatory particle AAA-ATPase 5a.

## Data Availability

Data sharing not applicable.

## Supplementary Materials

The supporting information can be downloaded at: https://www.mdpi.com/article/10.3390/ijms232214103/s1. References [167,168,169,170,171,172,173,174,175,176,177,178,179] are cited in the supplementary materials.
