# Supplementary material for: Identification of the Karyopherin Superfamily in Maize and Its Functional Cues in Plant Development
Source: Int J Mol Sci. 2022 Nov 15;23(22):14103. doi: 10.3390/ijms232214103 (PMC9699179; doi:10.3390/ijms232214103)
Supplement: Supplementary file 1 [file ijms-23-14103-s001.zip › ijms-1982330-supplementary.pdf]

Table S1. The Karyopherin Superfamily in *Arabidopsis thaliana*.

	Subfamily	Name (Aliases)	Gene ID	Function
Importin α	α1	IMPA1	AT3G06720	Flowering regulation [76]
		IMPA2	AT4G16143	Flowering regulation [76]; Plant immunity [167]
		IMPA3 (MOS6)	AT4G02150	Flowering regulation [76]; Cytokinin-activated cell division [121]; Plant immunity [168]
		IMPA4	AT1G09270	Stem cell differentiation [77]; Virulence protein nuclear import [169]
		IMPA5	AT5G49310	Unknown
		IMPA6	AT1G02690	Root development [78]; Cytokinin-activated cell division [121]
		IMPA7	AT3G05720	Unknown
		IMPA8	AT5G52000	Unknown
	PLANT α	IMPA9	AT5G03070	Root development [78]
Importin β	IMB1	ATKPNB1	AT5G53480	ABA-mediated drought response [41, 123]
	IMB2	ATTRN1	AT2G16950	miRNA activity regulation [89]; Circadian clock regulation [83]
	IMB3	KETCH1 (EMB2734)	AT5G19820	miRNA biogenesis [88]; Gametogenesis [170]
	IMB4	IMB4	AT4G27640	Root/ Ovule development [120, 171]; Cell wall assembly [82]
	IMB5	IMB5	AT1G26170	Unknown
	IPO8	AT3G59020	AT3G59020	Unknown
		SAD2 (GIR1)	AT2G31660	PCD process [92]; miRNA activity regulation [95]; ABA signal transduction [122]; UV-B response [172]; Trichome initiation [173]; Lignin modification [174]
	KA120	KA120	AT3G08960	Plant immunity [92]
	PLANTKAP	PLANTKAP	AT3G17340	Unknown
	TNPO3	MOS14	AT5G62600	Plant immunity [91]
		TNPO3	AT1G12930	Unknown
	XPO1	XPO1A (ATCRM1)	AT5G17020	Plant immunity [95]; ABA responses [124]; Chromatin regulation [139]; Heat response [175]; Gametophyte development [176]
		XPO1B (CRM1B)	AT3G03110	Plant immunity [90]; Gametophyte development [176]
	XPO2	XPO2	AT2G46520	Unknown
	XPOT	PAUSED (PSD)	AT1G72560	tRNA export [177, 178]
	XPO4	XPO4	AT3G04490	Plant immunity [179]
	XPO5	HASTY	AT3G05040	miRNA biogenesis [43,166]; Morphogenesis [103]
	XPO7	XPO7	AT5G06120	Unknown

Table S2. Distribution of Karyopherin genes in the Maize1 and Maize2 subgenomes.

Orthologous genes in Sorghum	Maize1 subgenome		Maize2 subgenome		Gene duplication	Dispersed genes in Maize	
	Name	Gene ID	Name	Gene ID		Name	Gene ID
Sobic.003G115100	ZmIMPα2	Zm00001d040274	ZmIMPα1	Zm00001d008345	R	ZmIMPα5	Zm00001d040153
Sobic.009G050700	ZmIMPα3	Zm00001d037606	ZmIMPα4	Zm00001d009850	R	ZmXPO5	Zm00001d009270
Sobic.009G102900	ZmIMB1c	Zm00001d038021	ZmIMB1d	Zm00001d010512	R	ZmPLANTKAPb	Zm00001d019335
Sobic.006G280000	ZmIMB2b	Zm00001d026696	ZmIMB2a	Zm00001d002936	R	ZmIPO8a	Zm00001d050526
Sobic.001G004400	ZmXPO1b	Zm00001d034914	ZmXPO1a	Zm00001d012815	R		
Sobic.001G115500	ZmXPO2a	Zm00001d033764	ZmXPO2b	Zm00001d013417	R		
Sobic.002G372500	ZmXPOTa	Zm00001d022125	ZmXPOTb	Zm00001d006845	R		
Sobic.001G219600	ZmTNPO3c	Zm00001d032699	ZmTNPO3b	Zm00001d014033	R		
Sobic.002G423200	ZmIMPα6	Zm00001d022536		/	L		
Sobic.008G135300	ZmIMB1a	Zm00001d030694		/	L		
Sobic.002G350600	ZmIMB3a	Zm00001d021893		/	L		
Sobic.002G350600	ZmIMB3b	Zm00001d021893		/	L		
Sobic.001G429600	ZmIMB4	Zm00001d028511		/	L		

Sobic.010G114900	ZmIMB5	Zm00001d045725	/		L
Sobic.004G145000	ZmIPO8b	Zm00001d016479	/		L
Sobic.005G222000	ZmPLANTKAPa	Zm00001d048628	/		L
Sobic.005G142800	ZmTNPO3a	Zm00001d052632	/		L
Sobic.001G219900	ZmXPO4	Zm00001d032704	/		L
Sobic.003G063000	/		ZmIMPα6	Zm00001d008640	L
Sobic.008G135200	/		ZmIMB1b	Zm00001d041556	L
Sobic.002G429300	/		ZmKA120	Zm00001d007225	L
Sobic.010G072800	/		ZmXPO7	Zm00001d037100	L

R for retained homoeolog gene, L for lost homoeolog genes.

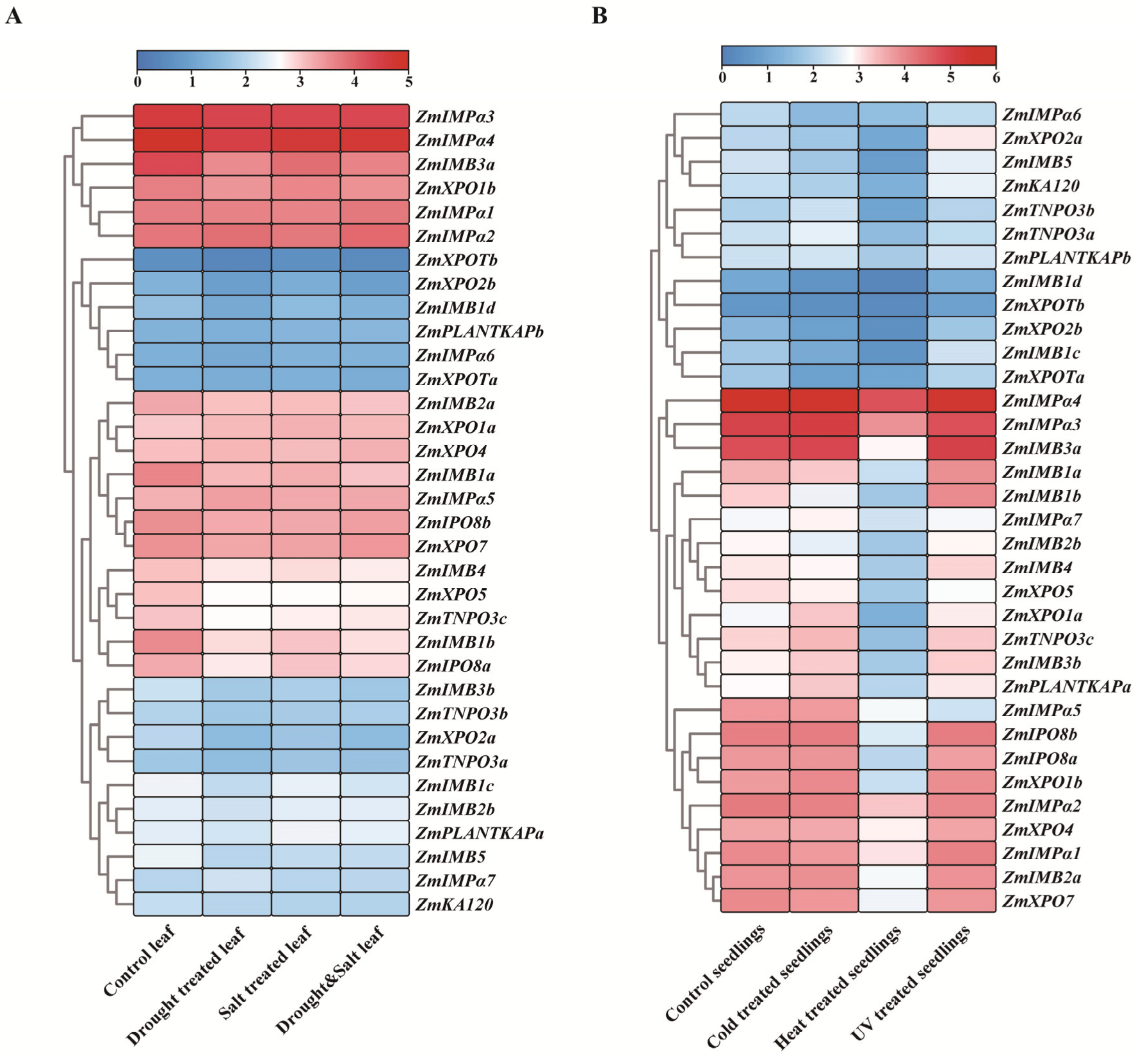

Figure S1 Heat-map of the expression profile of ZmKAP genes under abiotic stress. (A) The expression profile of *ZmKAP* genes under drought, salt, and drought & salt treatment. (B) The expression profile of *ZmKAP* genes under cold, heat, and ultraviolet (UV) treatment. The data comes from qTeller MaizeGDB

(https://qteller.maizegdb.org/). The expression value is calculated by log2 (TPM) and drawn by TBtools software.
